# Altered Placental Chorionic Arterial Biomechanical Properties During Intrauterine Growth Restriction

**DOI:** 10.1038/s41598-018-34834-5

**Published:** 2018-11-08

**Authors:** Shier Nee Saw, Jess Jia Hwee Tay, Yu Wei Poh, Liying Yang, Wei Ching Tan, Lay Kok Tan, Alys Clark, Arijit Biswas, Citra Nurfarah Zaini Mattar, Choon Hwai Yap

**Affiliations:** 1 Department of Biomedical Engineering, National University of Singapore, Singapore, Singapore; 2 Department of Obstetrics and Gynecology, Yong Loo Lin School of Medicine, National University of Singapore, National University Health Systems, Singapore, Singapore; 3 Department of Obstetrics & Gynecology, Singapore General Hospital, Singapore, Singapore; 4 Auckland Bioengineering Institute, University of Auckland, Auckland, New Zealand

## Abstract

Intrauterine growth restriction (IUGR) is a pregnancy complication due to placental dysfunction that prevents the fetus from obtaining enough oxygen and nutrients, leading to serious mortality and morbidity risks. There is no treatment for IUGR despite having a prevalence of 3% in developed countries, giving rise to an urgency to improve our understanding of the disease. Applying biomechanics investigation on IUGR placental tissues can give important new insights. We performed pressure-diameter mechanical testing of placental chorionic arteries and found that in severe IUGR cases (RI > 90^th^ centile) but not in IUGR cases (RI < 90^th^ centile), vascular distensibility was significantly increased from normal. Constitutive modeling demonstrated that a simplified Fung-type hyperelastic model was able to describe the mechanical properties well, and histology showed that severe IUGR had the lowest collagen to elastin ratio. To demonstrate that the increased distensibility in the severe IUGR group was related to their elevated umbilical resistance and pulsatility indices, we modelled the placental circulation using a Windkessel model, and demonstrated that vascular compliance (and not just vascular resistance) directly affected blood flow pulsatility, suggesting that it is an important parameter for the disease. Our study showed that biomechanics study on placenta could extend our understanding on placenta physiology.

## Introduction

Intrauterine growth restriction (IUGR) is a condition where high vascular resistance in the placenta results in insufficient oxygen and nutrients transferred to the fetus, leading to reduced fetal growth. This condition leads to 5–10 times higher mortality risks, and long-term complications such as neurodevelopment impairment, hypertension, and diabetes^1–3^, representing the second foremost cause of perinatal morbidity and mortality^4^. The prevalence of IUGR is approximately 3% in developed countries and can reach up to 15% in developing countries^5^. The most common cause of IUGR is placental insufficiency, where there is a reduction in the density and size of blood vessels^6^ leading to increased vascular resistance^7^, and thus reduced oxygen and nutrient exchange. To date, there is no proven strategy to prevent or treat IUGR^8^, although well-timed early delivery of the baby to avoid *in utero* hypoxia and malnutrition is shown to improve outcomes^9^. Therefore, improved knowledge of this disease is urgently needed for the development of diagnostic, management and treatment strategies.

Biomechanics analysis of placental tissues can be one new way of understanding IUGR. In the past, biomechanics studies of blood vessels have developed important knowledge on vascular growth and remodeling process in response to biomechanical force stimuli^10,11^, and have played important role in the development of tissue-engineered blood vessels^12–14^. Biomechanics analysis of normal versus diseased tissues have also deepened our understanding on tissue properties changes during disease states^15–17^ and have led to the advent of employing elastography (non-invasive measurement of tissue stiffness) as a diagnosing tool to detect diseases^18,19^. We have recently performed studies of mechanical properties of normal and IUGR placental tissues^20,21^, while others have demonstrated the use of elastography in detecting placental diseases^22,23^. Furthermore, 1D biomechanics modeling of vascular network flows has enabled a better understanding of transport phenomenon and temporal variability of flow^24,25^. In the current study, we applied these biomechanical analyses to placental chorionic arteries, based on a belief that they can generate much more new insights on IUGR disease.

Blood flowing into the placenta via the umbilical artery is pulsatile, and its pulsatility can be quantified by Doppler indices, such as resistance index (RI) and pulsatility index (PI). These Doppler indices are often thought to directly represent the downstream placental vascular resistance and elevate when placental resistance increases^26,27^ and that increased placental resistance generally occurs with a substantial reduction of vascular size and density^28^. As such, umbilical Doppler indices are routinely used for risk stratification, surveillance, and obstetric management once an IUGR fetus is identified by ultrasound biometry measurements. This was supported by observations that absent or reversed end-diastolic flow in the umbilical arteries were mostly found in very sick IUGR cases^29,30^. However, recent PORTO multicenter study reported that only 46% of IUGR cases demonstrated significantly enhanced PI (>90^th^ centile)^31^, implying that the RI and PI to be unreliable in representing placental vascular resistance. It might be the case that other factor, such as vascular compliance/distensibility, played a role in affecting the blood pulsatility, as proposed by previous *in vitro* experiments^32^.

Further, histological results had shown that IUGR placenta exhibited reduced elastin tissues fibers^33^, and direct mechanical testing of placenta showed that stiffness of placenta was different between normal and IUGR^20^. Thus, it is likely that the compliance of placental blood vessels could also have altered during disease and that is only in some disease cases where these mechanical properties changes were significant enough to influence the RI and PI values. Unfortunately, to date, there has been no study on the vascular mechanics or compliance of placental arteries either in normal or in IUGR pregnancies to test these hypotheses.

In this study, we have also performed numerical simulation of placental blood flow to supplement our experimental results. The two-element Windkessel mathematical model has been widely used for accurate simulation of vascular pressure and blood flow within vessels based on the interaction between stroke volume and arterial compliance^24,34^. This model has been applied to the pulmonary vascular bed and placenta^27,35^ and is useful to understand the effect of vascular resistance and compliance on blood flow, especially in diseased state, in which alterations in vascular resistance and compliance are anticipated^34,36^. We adopted this model in our study to investigate the relationship between the flow pulsatility and vascular compliance and resistance using clinically measured umbilical Doppler waveforms.

The overall objective of this study is to characterize the biomechanical, anatomical and hemodynamic properties of placental chorionic vessels in normal and IUGR placentae using a host of biomechanical techniques, including mechanical testing, constitutive modeling, 1D flow modeling, and corrosion casting. Substantial focus was placed on detecting changes to vascular distensibility during disease, and the role it played in affecting flow patterns and Doppler indices.

## Methods

All placental tissues were obtained from women who delivered at National University Hospital and Singapore General Hospital, Singapore, in accordance with approved guidelines at the hospital. All human sample testing protocols were approved by the Singapore National Healthcare Group Domain Specific Review Boards and SingHealth Centralised Institutional Review Board, and informed consent was obtained from all participants. Following delivery, placentae were immediately placed in Phosphate Buffered Saline (PBS) and were kept in the refrigerator when not being handled. All experiments were conducted within three days after delivery. According to a previous study, properties of extracted arteries would remain unchanged in three days^37^.

### Placental Chorionic Arteries Experiment

#### Anthropometric Analysis

To study the placental chorionic vascular patterns and anatomical characteristics, we performed vascular corrosion casting on 10 normal and 4 IUGR post-delivered placentae. IUGR was defined as antenatal estimated fetal weight (EFW) measured via ultrasound fell below the 10^th^ centile, further serial monitoring did not show improvement or showed worsening EFW. Among these IUGR cases, none had umbilical arterial RI or PI above the 90^th^ centile. Vascular corrosion casting was performed using similar protocols adopted in the past studies^38,39^. Dried placental vascular cast obtained after corrosion casting procedure was scanned with Siemens Dual Source Definition FLASH 256 computed-tomography (CT) scanner with the X-ray tube set at 120 kV, 40 mA, convolution kernel at J35, slice thickness of 0.5 mm, and pixel spacing of 0.2–0.4 mm. 3D segmentation of vascular cast was performed using an open source program, Vascular Modelling Toolkit (VMTK)^40^, which could also provide the cross-sectional areas and centerline coordinates of vessels. Placental chorionic vessels anthropometric parameters, such as vascular radius, branching angle and branching ratio were computed. The details of vascular corrosion casting procedures and equations used to compute anthropometric parameters can be found in the Supplementary Document.

#### Pressure-Diameter Mechanical Testing

To study the mechanical properties of placental chorionic arteries, we collected additional 6 normal and 10 IUGR placentae. Among the 10 IUGR placentae, 4 of them had abnormal umbilical arterial blood flow (RI/PI > 90^th^ centile). As such, we divided the IUGR into two groups, which were IUGR (low EFW, normal RI/PI with values <90^th^ centile) and severe IUGR (low EFW, high RI/PI with values >90^th^ centile).

**Figure 1:**
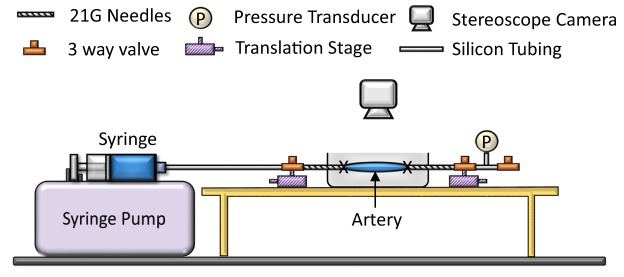
Experiment setup for mechanical testing of placental chorionic arteries. PBS solution was injected slowly into the vessels until pressure reached 40 mmHg. Diameter and pressure changed during pressurization were recorded for analysis.

Mechanical testing of placental chorionic arteries was performed using a custom experiment setup as shown in Fig. 1. Details of ways in extracting the placenta arteries and pressure diameter experiment can be found in the Supplementary Document. In brief, 18 normal, 17 IUGR and 13 severe IUGR placental chorionic arteries from 6 normal and 6 IUGR and 4 severe IUGR placentae were extracted and tested. The breakdown of the vessels for each placenta: 3 vessels from 6 normal placentae each, 3 vessels from 6 IUGR placenta each, however, one IUGR placenta had only 2 vessels tested because one vessel leaked during the experiment, thus it was eliminated from the analysis, resulting in only 17 vessels. As for severe IUGR, the first 3 placentae had 3 vessels extracted and tested and the last placenta had 4 vessels tested, resulting in 13 vessels. The extracted vessel had a length of 20 mm. The vessels mostly retracted to around 18 mm after extraction and were stretched back to its original length of 20 mm on the testing rig to mimic *in vivo* conditions, giving an initial axial stretch of 1.1. With the three-way valve at the rightmost point closed, PBS fluid was injected into the vessels with a constant rate of 90 μl/min until it reached 40 mmHg. Thereafter, the three-way valve was opened to release the luminal pressure back to atmospheric pressure. This process of pressurization and de-pressurization was repeated for eight cycles - the first five cycles were used for pre-conditioning and the last three cycles were used for analysis. The vessel was imaged using a CMOS camera (XCAM1080PHA, Touptek) and the diameter changed of the vessel was tracked over time with a custom written MATLAB code.

The distensibility of the artery at specific luminal pressure was computed using Equation 1.

1 $${Distensibility}\,(mmH{g}^{-1})=\frac{{\rm{d}}\lambda }{{\rm{d}}P}$$

where *λ* is the circumferential stretch (ratio of final diameter to initial diameter) and *P* is the vascular luminal pressure.

After the testing, placental chorionic arteries were processed for Verhoeff-van Gieson (VVG) stain to evaluate their elastin and collagen contents. For each stained slide, three images were taken, and the results for these triplicates were averaged before being reported. From the microscopic images of histology slides, ImageJ was used to measure the relative abundance of collagen and elastin by computing the number of pixels stained in pink/purple (collagen) and in black (elastin).

#### Constitutive Modelling of Chorionic Vascular Mechanical Properties

To obtain an accurate description of the mechanical properties, the stress-free state of the arteries must be considered^41,42^. Short ring segment of the placental chorionic artery was extracted. Thickness and diameter of the ring vessel were measured. Then, a radial cut was imposed on the ring vessel, resulting in an open C sector due to the release of pre-stress. After the radial cut, the vessel was incubated for four hours at 37 °C to allow the vessels achieved their stress-free configuration fully. ImageJ was used to measure the opening angle (Figure S1 in Supplementary Document). The opening angle referred to the angle between the two lines that connect from the center of the inner sector to the two ends of the inner sector.

The placental chorionic artery was modelled as incompressible, orthotropic and homogeneous thick wall cylindrical tube. The vascular mechanics of placental chorionic arteries were characterized using the pseudo-strain energy function (Equation 2)^42^, named as Fung model. We incorporated residual stress in our model, assumed negligible cross stretch (*b*_4_ = *b*_5_ = *b*_6_ = 0) and similar stiffness in all three direction (*b*_1_ = *b*_2_ = *b*_3_). Detailed analytical framework can be found in the Supplementary Document.

2 $${W}({E})=\,\frac{{c}}{2}({e}^{{{b}}_{1}{{E}}_{{\theta }}^{2}+{{b}}_{1}{{E}}_{{z}}^{2}+{{b}}_{1}{{E}}_{{r}}^{2}}-1)$$

where *c* and *b*_1_ are the material parameters, *E* is the Green Strain and the subscripts of *E* refer to the three orthogonal axes.

The material parameters were obtained by performing curve-fitting using the built-in Nelder-Mead algorithm in MATLAB. The goodness-of-fit was evaluated by the coefficient of determination, R^2^ value.

### Statistical Analysis

Given the repeated measurements within a placenta, a regression model was used to fit our results using generalized estimating equation (GEE) method in R, using *geepack* package^43^. The model was fitted with the individual specimens grouped according to placenta and then fetus status (Normal/IUGR/Severe IUGR). The used of GEE method accounts for both repeated measurements and different sample sizes within each placenta^44^ and such method had been used in others studies with similar situation^45,46^.

To compare the difference in parameters between normal and diseased groups, a full model was compared with a restricted model where the explanatory variable (fetus status) was removed. If P-value of the fetus status’ coefficient was less than 0.05, it suggested that fetus status played a significant effect in affecting the response variables. To do this, a*nova* method in *geepack* package was used. When comparing the difference in parameters among the three groups – normal, IUGR and severe IUGR, similar procedure was done. If the fetus status played a significant role in affecting the response variable, post hoc test was carried out to confirm where the differences occurred between groups by testing the difference in the regression coefficients using *contrast* method. Bonferroni adjusted p-value, which counteracted the problem of multiple comparisons, were reported. The data was said to be significantly different if P < 0.05.

### 1D Modeling of Blood Flow in Umbilical-Placenta Vasculature

**Figure 2:**
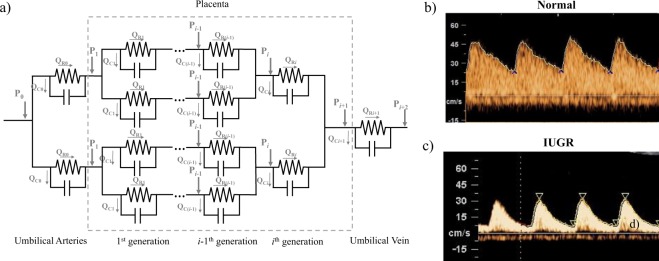
(**a**) Schematic of the Windkessel modeling. The umbilical-placenta circulation is modeled as a series of two-element Windkessel models. *P*_*i*_ – pressure at *i*^*th*^ generation vessel; *Q*_*Ri*_ – Flow through the *i*^*th*^ resistance; *Q*_*Ci*_ – rate of fluid going into compliance storage at the *i*^*th*^ generation vessel. (**b**,**c**) Clinical pulse wave Doppler velocity of (**b**) normal and (**c**) IUGR umbilical artery, which were used in the Windkessel modeling.

A network of two-element Windkessel models (Fig. 2) was adopted to model the blood flow in the placental vasculature and to investigate the effect of placental vascular resistance and compliance on umbilical arterial flow parameters such as mean flow rate, RI, PI and peak backflow rate. Each vessel consisted of one resistor and one capacitor, which represented the vascular resistance and compliance respectively. The model consisted of 1 generation of umbilical artery, 10 generations of placental arteries, 1 generation of microvilli, 10 generations of placental veins and 1 generation of umbilical vein^47^. The details of equations and placental vasculature measurements used in the model can be found in Supplementary Documents.

Umbilical arterial pulse pressure range was set at 50/25 mmHg (systolic/diastolic) in normal^48^ and 80/25 mmHg in IUGR^49^, while umbilical venous pressure was prescribed as a time-independent constant at 5.3 mmHg in normal and IUGR cases^50^. The pressure versus time waveform shape was set to be similar as the velocity versus time waveform plot, obtained from clinical pulse wave Doppler measurements, one from normal and one from IUGR with RI > 90^th^ centile (Fig. 2b,c).

The model was first adjusted by varying the umbilical-placental resistance and compliance such that the calculated output flow rates matched the sonographically recorded flow rates. After the fitting, the umbilical-placental resistance and compliance for normal and IUGR can be obtained. Subsequently, using the normal fetus’s condition as the baseline, a parametric test was conducted by applying one single multiplier to the placental resistance and compliance to observe their effects on the flow parameters. The flow rates in the umbilical-placental circulation were computed using the built-in Ordinary Differential Equation (ODE) solver in MATLAB® (Mathworks Inc., Natick, USA).

## Results

Patients’ characteristics recruited in this study are tabulated in Table S2 in the Supplementary Document. IUGR and severe IUGR babies were significantly lighter than normal babies at birth (P = 0.00027). No significant difference was observed in RI and PI between the normal and IUGR babies.

### Placental Chorionic Arteries Experiment

#### Anthropometric Analysis

**Figure 3:**
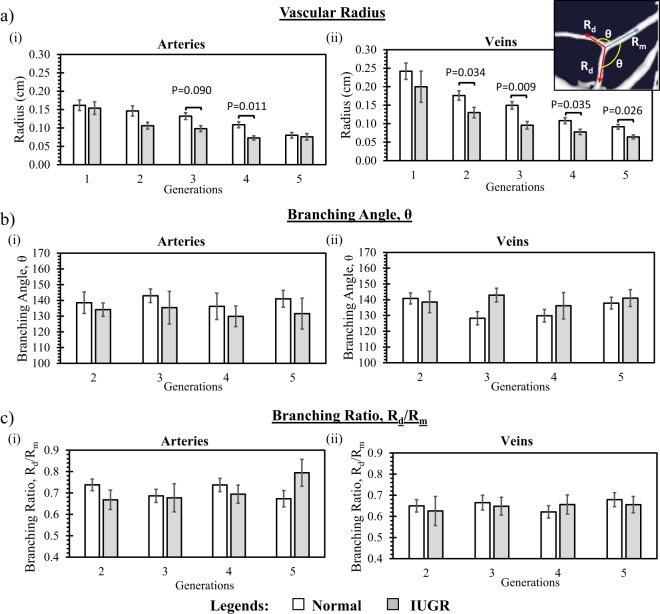
(**a**) Placental vascular radius, (**b**) branching angle and (**c**) branching ratio (daughter vessel radius/mother vessel radius) computed from 10 normal and 4 IUGR placentae. Data presented are mean and standard errors of measurements. Vessels at 1^st^ generation refer to vessels after branching from umbilical vessels.

Figure 3 shows the geometrical measurements of the placental vasculature at different vascular generations. 1^st^ generation of vessels referred to the vessels after branching from umbilical vessels, similar annotation as Fig. 2a, defined as the number of times vascular branching occurred before blood from the umbilical artery reached a particular vessel. Results showed that our geometrical measurements in normal placentae were in qualitative agreement with previous study^39^. Further, vascular sizes of the IUGR placental veins were substantially smaller than those in the normal placenta at most of the vascular generations investigated (P < 0.05). As for placental arteries, IUGR arteries generally had smaller vascular size but the difference was only significant at the 4^th^ generation and weakly significant at the 3^rd^ generation. Arterial and venous branching angles and daughter-to-mother vascular radius branching ratio, however, were generally very similar across the various vascular generations.

#### Mechanical Properties of Placental Chorionic Arteries

**Figure 4:**
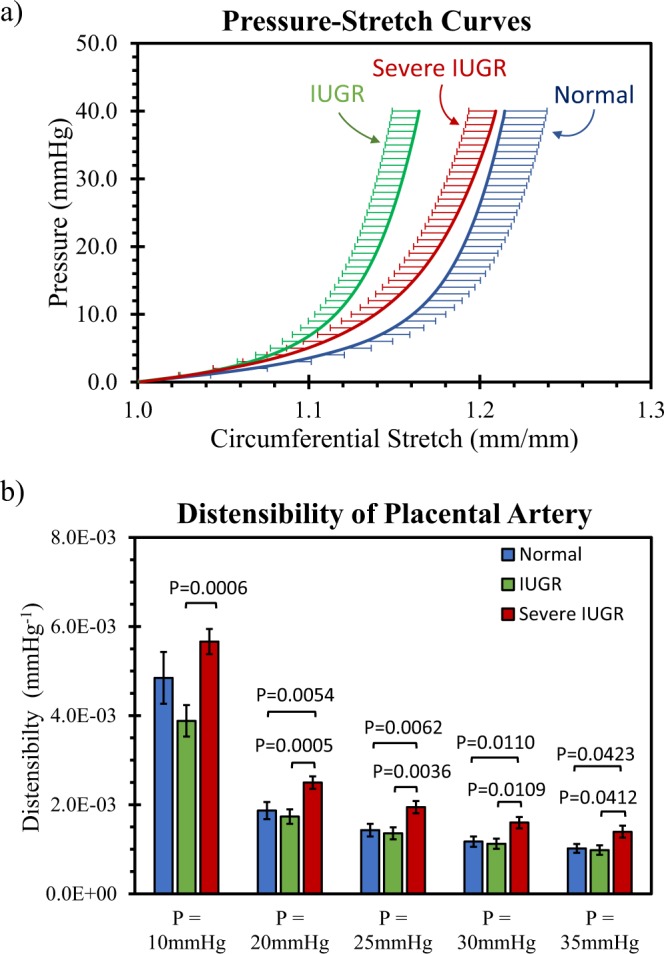
(**a**) The average mechanical response curves from mechanical testing. The three curves represent the data averaged from 18 normal, 17 IUGR and 13 severe IUGR placental chorionic arteries respectively. (**b**) Distensibility of placental chorionic arteries, computed from reciprocal of the pressure-stretch gradient, at different luminal pressures for normal, IUGR and severe IUGR groups. Data presented are mean and standard error.

The mean pressure-circumferential stretch curves of the placental chorionic artery for normal, IUGR (RI/PI < 90^th^ centile) and severe IUGR (RI/PI >90^th^ centile) are plotted in Fig. 4a. Normal and IUGR placental chorionic arteries had pressure-stretch curves with similar shapes but the IUGR curve was shifted to the left. As for severe IUGR, the arterial pressure-stretch curve had a gentler gradient than the other groups. The reciprocal of the gradient of the pressure-stretch curve at specific luminal pressure represented the arterial distensibility at that particular pressure. Figure 4b illustrates the distensibility of the placental chorionic arteries at different luminal pressures. Results demonstrated that the distensibility of the severe IUGR artery was significantly higher than the normal and IUGR arteries at all luminal pressures, except at 10 mmHg when compared with the normal artery.

Vascular geometric analysis showed that no significant difference among the three groups in opening angle (Normal: 91.48° ± SD32.61°; IUGR: 106.45° ± SD 40.27°; Severe IUGR: 96.88° ± SD 36.43°, P = 0.49) and thickness/diameter ratio (Normal: 0.586 ± SD 0.131; IUGR: 0.616 ± SD 0.227; Severe IUGR: 0.654 ± SD 0.165, P = 0.75). It is noteworthy that our opening angle measurements for the normal placental chorionic arteries had similar magnitudes as those on carotid arteries in other studies^41,51^.

**Figure 5:**
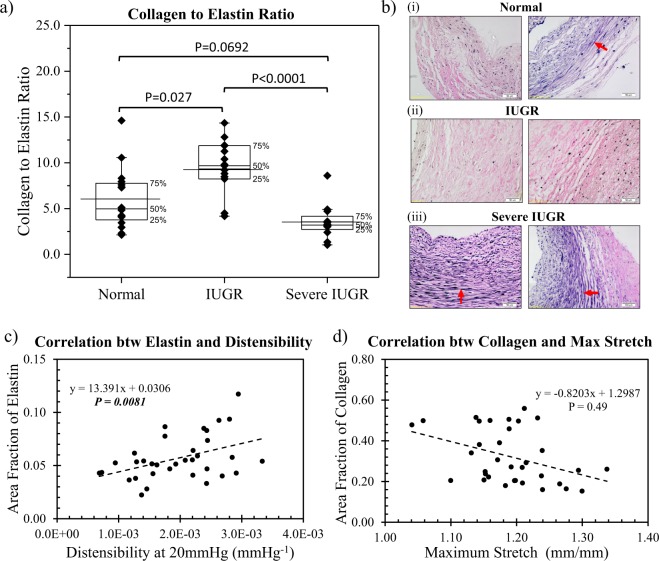
(**a**) Box plot showing individual collagen to elastin ratio and the mean in normal, IUGR and severe IUGR placental chorionic arteries. (**b**) Representative Verhoeff-van Gieson stain images for normal, IUGR and severe IUGR. Collagen was stained pink/purple while elastin was stained black, as indicated by red arrows. (**c**,**d**) Regression analysis between (**c**) area fraction of elastin and distensibility at 20 mmHg and (**d**) area fraction of collagen and maximum stretch obtained from the pressure-stretch curve. P indicates the significance of the regression line coefficient using the GEE method.

Figure 5 shows the collagen to elastin ratio and representative VVG stain images for each group. From the images, we could qualitatively observe that elastin (stained in black) was most abundant in severe IUGR arteries as compared to normal and IUGR arteries. After image analysis, severe IUGR group was found to have the lowest collagen to elastin ratio (C/E = 4.00 ± SD 1.61), followed by normal (C/E = 6.33 ± SD 3.18) and IUGR (C/E = 11.17 ± SD 4.49). The reduction of collagen to elastin ratio in severe IUGR arteries might explain the increased arterial distensibility, as shown in Fig. 4. Regression analysis corroborated with this notion as we observed statistically significant correlations between elastin quantity and distensibility (Fig. 5c). However, no significant correlation was observed between collagen quantity and maximum stretch (Fig. 5d).

The Fung hyperelastic mechanical model was able to fit the experimental datasets very well, with R^2^ value ≈ 1. The material parameters obtained from the model are summarized in Table 1. Severe IUGR showed significantly larger parameter *c* (P = 0.0293) and smaller parameter *b*_1_ (P = 0.0271) as compared to IUGR.

**Table 1:** Material parameters determined from normal, IUGR and severe IUGR placental chorionic arteries using Fung model.

	Normal (n = 18)	IUGR (n = 17)	Severe IUGR (n = 13)
*c*	125.82 ± 41.58	99.91 ± 39.08	**345.65 ± 100.83** ^*^
*b*_1_	20.63 ± 4.57	26.83 ± 7.52	**8.56 ± 1.36** ^**†**^
R^2^	0.9990 ± 0.0010	0.9988 ± 0.0018	0.9885 ± 0.0019

Values indicated are mean and standard errors. *P = 0.0293 vs IUGR, ^†^P = 0.0271 vs IUGR. R^2^ refers to the coefficient of determination.

### 1D Modeling of Blood Flow in Umbilical-Placenta Vasculature

**Figure 6:**
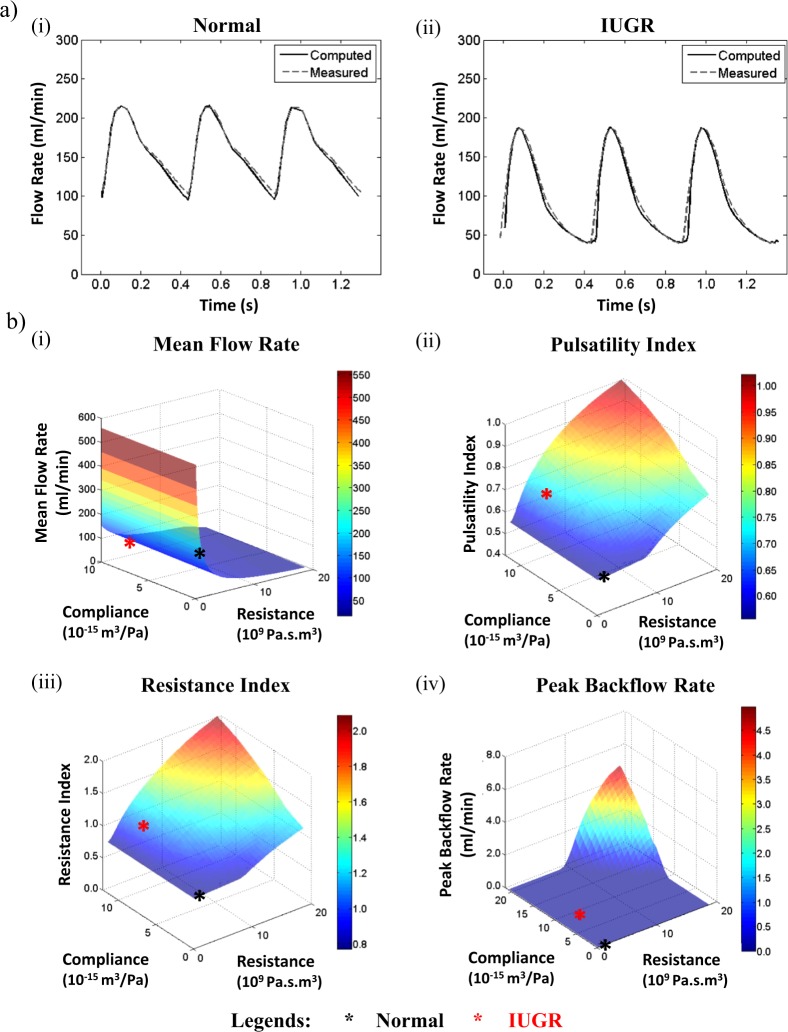
1D Modeling Results. (**a**) Clinically measured and modelled umbilical arterial blood flow waveforms showed a good fit in both normal and IUGR after tuning the umbilical-placental compliance and resistance in the Windkessel model. Their umbilical-placental resistance, compliance and other flow parameters were plotted as black (Normal) and red (IUGR) asterisks in (**b**). (**b**) Effect of umbilical-placental resistance and compliance on (i) mean flow rate, (ii) PI, (iii) RI, and (iv) peak backflow rate.

Figure 6a shows that the umbilical arterial blood flow waveforms from the Windkessel model could be tuned to match the clinical ultrasound measurements well. Subsequently, the vascular resistance and compliance were arbitrarily set at various values within a bounded range and the simulation was re-run to understand their effects on umbilical flow characteristics, and results are shown in Fig. 6b. We observed that mean flow rate had an inverse relationship with resistance but did not change with compliance, but RI and PI increased non-linearly with increasing resistance or compliance. RI and PI were shown to be dependent on both resistance and compliance, indicating that resistance alone could not uniquely represent them. The same RI and PI read-outs, as represented by the same color on the contour surface plots, could be obtained with different placental resistance values via adjusting the placental compliance value. This suggested that the prevailing assumption that these Doppler indices represent directly the downstream placental vascular resistance was likely to be erroneous. The peak backflow rate, which was the highest amount of volumetric flow rate in the retrograde direction that occurred usually at the end-diastolic time point, was non-zero only when resistance and compliance were sufficiently high. In the modelling of normal (black asterisk) and IUGR (red asterisk) pregnancies, the overall umbilical-placental vascular resistance and compliance had to be higher in IUGR to achieve a good fit between modelled and measured flow waveforms. This suggested that the increased RI and PI were partially due to increased vascular compliance.

## Discussion

The placenta is an important organ that supports the fetus growth throughout the pregnancy, and placenta diseases can be devastating to both fetal and mother’s health, but our knowledge of placenta physiology remains very limited. From the literature, it is apparent that tissue biomechanical characteristics and physiology has an intricate cause and effect relationship. Numerous literatures reported different pathological conditions such as diabetes^52^, hypertension^53,54^, smoking^55^ and obesity^56^ resulted in altered arterial mechanical properties. On the other hand, biomechanical forces and tissue stiffness are known to influence pathogenesis and disease progression. For example, low and oscillatory fluid mechanical shear stresses were believed to cause atherosclerosis^57^, and biomechanical forces in tumor were believed to influence tumor growth^58^. For this reason, we advocate studies of the placenta and its diseases from a biomechanics point of view.

To date, there is little information on the mechanical properties of human placental chorionic arteries in normal or IUGR pregnancies. This motivated our study, and our results revealed an interesting finding that the severe IUGR placental chorionic arteries exhibited significantly higher vascular distensibility, which was likely a result of microstructural changes to the blood vessels’ walls, and which would likely contribute to the increased umbilical Doppler indices.

From our mechanical testing results, we noted that the placental chorionic arteries from all three groups exhibited similar typical mechanical testing pressure-stretch curves with a low stiffness at a low stretch and a high stiffness at a high stretch. However, the severe IUGR artery had significantly higher distensibility at pressures between 20 mmHg to 35 mmHg, suggesting that at these physiological pressures, the vessel would undergo higher periodic stretch and luminal size changes if exposed to the same pulse pressure.

We believe that the observation of increased vascular compliance in severe IUGR vessels is an important one because umbilical flow Doppler indices, RI and PI, are used routinely in detecting IUGR. Increased umbilical flow Doppler indices in diseased cases are often thought to be due to the increased vascular resistance of blood vessels in the placenta and hence these indices are always used as direct indicators of placental vascular resistance^59,60^. However, our mechanical testing results showed that chorionic vessels from the severe IUGR cohort, with RI or PI above the 90^th^ centile, had higher compliance than normal and IUGR (RI/PI < 90^th^ centile) vessels. Our mathematical model corroborated with this finding, demonstrating that, on top of vascular resistance, vascular compliance also affected the RI and PI values, and that increased compliance was necessary to match the modelled and clinically measured umbilical flow waveforms in IUGR case. Further, reversed flow would not occur if the vessel had low compliance. This suggested that high RI, PI and/or reversed end-diastolic blood flow observed in IUGR umbilical flow was at least partially due to compliance changed, in addition to resistance changed. To date, however, evaluation of vascular resistance has dominated the literature on IUGR vasculature and hemodynamics^61–64^, and little attention has been paid to arterial compliance.

There are several factors to consider why severe IUGR vessels become more distensible. Previous studies have found that hypoxic conditions can increase the distensibility of the uterine arteries^65^. This corroborates with our finding that the severe IUGR placental chorionic arteries have generally increased distensibility, raising the possibility that in severe IUGR cases, due to insufficient oxygen transfer from the mother to the fetus, placental vessels experience low oxygen environment and develop increased distensibility. The fact that the placental chorionic arteries are among the arteries with the lowest oxygen content in the fetal circulation further support this hypothesis.

Previous study showed that IUGR (with high umbilical RI) umbilical artery exhibited a reduction of vascular compliance^66^, which stood in contrast to our observation that the arterial distensibility/compliance increased in severe IUGR group. One possible explanation for this is that this increased vascular stiffness/decreased vascular compliance could be a response to the elevated systemic pressure that is experienced by IUGR fetuses^67^ and that the umbilical arteries are more susceptible to pressure changes caused by systemic pressure elevations since they are closer to the heart than the placental arteries. Other studies have also reported an increase in vascular stiffness (or decreased of vascular compliance) in carotid and umbilical arteries in IUGR rats and ewes^68–70^. However, it was not known whether the IUGR fetus in these studies had normal or abnormal umbilical arterial blood flow, and thus comparison with our results could not be made.

From the mechanical testing and constitutive modeling results, there were clear differences between the two groups of severe IUGR and IUGR. As such, the changes from the group of normal to IUGR cannot be extrapolated to severe IUGR. Further, Fig. 5a showed that the IUGR vessels exhibited greater collagen to elastin ratio than the normal vessels, but the severe IUGR vessels exhibited lower collagen to elastin ratio than the normal vessels. Together, these results raised the possibility that the IUGR and severe IUGR groups could be different disease manifestations, a notion that warrants future investigations, as it might have clinical implications.

Apart from these differences, other investigations did not show a difference between normal and IUGR groups. Our vascular geometrical analysis showed that the vascular branching patterns, opening angle and thickness/diameter ratio were very similar in both normal and IUGR placentae at a macro level, suggesting that the diseased placenta retained some features from the normal placenta and might have similar vascular growth and remodeling responses and transport mechanisms as the normal placenta. This could be corroborated by one of our previous studies showing that the umbilical vascular flow mechanical force environments between normal and IUGR were very similar^71,72^.

There were a few limitations in this study. Firstly, severe IUGR placentae were not collected for anthropometric analysis. This was because there were few severe IUGR cases and sample collection was difficult. However, we believe that the vascular size in severe IUGR placenta will be significantly smaller as it represents the more severe disease case. Secondly, the pressure-diameter mechanical testing experiments were conducted at room temperature instead of body temperature but previous studies had shown that the effect of temperature on the arterial mechanical behavior was small^73^.

In conclusion, we had adopted various biomechanical analyses to study the IUGR placenta and revealed essential differences between normal and IUGR placental vessels. Our results showed that severe IUGR arteries had greater vascular distensibility as compared to normal and IUGR arteries, which could explain the clinical observation of abnormally high blood flow pulsatility in these severe IUGR umbilical cords. Mathematical modelling of blood flow in the umbilical-placenta vascular network corroborated these results and demonstrated that vascular distensibility to be an important determinant of blood flow pulsatility. The alteration of vascular mechanical properties in arteries in diseased placenta could be due to the observed changes in their extracellular matrix. The insights provided by this study demonstrated that biomechanical analysis could be a relevant and useful technique in exploring the placenta physiology.

## Electronic supplementary material

Supplementary Information

## Data Availability

The datasets generated during and/or analyzed during the current study are available from the corresponding author on reasonable request.
