# Supplementary material for: Altered Placental Chorionic Arterial Biomechanical Properties During Intrauterine Growth Restriction
Source: Sci Rep. 2018 Nov 8;8:16526. doi: 10.1038/s41598-018-34834-5 (PMC6224524; doi:10.1038/s41598-018-34834-5)
Supplement: Supplementary file 1 — Supplementary Information [file 41598_2018_34834_MOESM1_ESM.docx]

SUPPLEMENTARY DOCUMENT

**TITLE: ALTERED PLACENTAL CHORIONIC ARTERIAL BIOMECHANICAL PROPERTIES DURING INTRAUTERINE GROWTH RESTRICTION**

*Shier Nee Saw, Jess Jia Hwee Tay, Yu Wei Poh, Liying Yang, Wei Ching Tan, Lay Kok Tan, Alys Clark, Arijit Biswas, Citra Nurfarah Zaini Mattar, Choon Hwai Yap*

**METHODS**

(A) Placental Chorionic Arteries Experiment

*Vascular Corrosion Casting Procedures*

Post-delivered placentae were flushed with phosphate-buffered saline (PBS) from the umbilical vein until the effluent fluid was clear and the vascular bed appeared blood-free. Then, the placental vasculature was perfused with radiopaque methylmethacrylate-based casting polymer material using Batson #17 Anatomical Corrosion Kit (Polysciences Inc. Europe) or Unifast Trad dental polymer (Unifast Trad, GC Dental Co, Tokyo, Japan), which were used in the past corrosion casting studies ^1,2^**.**  The casting material was always freshly prepared and mixed with blue or red dyes for arteries and veins respectively before being injected into the placental vasculature via umbilical vessels until back pressure prevented further injection. The umbilical cord was then clamped to prevent leakage and the whole placenta was stored in a refrigerator at a temperature of 4°C for more than 24 hours until the polymer hardened. Finally, the placenta with the hardened polymer was immersed in 40% potassium hydroxide solution (KOH; Alfa Aesar, Massachusetts, United States) with a temperature of 70°C for tissue corrosion. This tissue corrosion was repeated with clean KOH solution until all placental tissue was completely corroded. Thereafter, the placental vascular cast was rinsed with water and left to dry.

*Geometrical Analysis*

The radius of the vessel was back computed from the cross-sectional area assuming a perfectly circular cross-section. To compute branching angle, line vectors of mother and daughter branching vessels were first obtained. These line vectors were taken as the tangent to a short segment of a vessel at its bifurcation point. Branching angle, *θ* was computed using dot product between the mother and daughter vessels’ vectors (Eq. S1).

$\boldsymbol{\theta=}\mathbf{cos}^{\mathbf{-1}} \frac{\boldsymbol{V}_{\boldsymbol{m}}\boldsymbol{\cdot}\boldsymbol{V}_{\boldsymbol{d}}}{\left| \boldsymbol{V}_{\boldsymbol{m}} \right|\boldsymbol{|}\boldsymbol{V}_{\boldsymbol{d}}\boldsymbol{|}}$ ***Equation S1***

where *V_m_* refers to the mother vessel’s vector and *V_d_* refers to the daughter vessel’s vector.

Branching ratio was computed as the radius ratio between the daughter's vessels to mother’s vessels.

*Pressure-Diameter Mechanical Testing*

Placentae were flushed with PBS solution via the umbilical vessels to remove the remnant blood in the vascular bed. Placental arteries were located by their bulge as PBS solution was injected into the umbilical artery. Placental arteries were extracted carefully with two razor blades attached to each other and separated by a fixed distance of 20 mm. After the extraction, the arteries contracted slightly due to residual stresses. With each placental artery extraction, the directly adjacent segment (approximately 7 mm in length) was also harvested for vascular geometry and opening angle quantifications. According to the previous study, properties of extracted arteries would remain unchanged within three days ^3^, as such, all experiments were conducted within three days of harvest and were kept in the refrigerator when not being handled.

Mechanical testing of the placental arteries was performed using a custom experiment setup (Fig. 1 in manuscript). The two ends of the artery were mounted on 21G needles using 5-0 or 6-0 sutures, depending on the size of the artery. Excess tissues around the vessel were carefully removed. Vessels were mostly retracted to around 18 mm after extraction, but the vessels were stretched back to its original length of 20 mm on the testing rig to mimic *in vivo* conditions, giving an initial axial stretch of 1.1. This was accomplished by moving the needles (with the artery sutured to it) apart using the micrometer-controlled translational stage (MT1/M Thorlabs Inc., NJ, USA). This stretch ratio was maintained for the rest of the experiment. At one end of the artery, a syringe pump (SPLab01, Shenzhen Precision Pump Co. Ltd, China) was used to inject PBS into the vessel. Pressure of the vessel was measured using a pressure transducer (MLT844, ADInstruments, Australia) that was connected to the other end of the artery. PBS was dyed blue using commercial food dye to detect any leakage and enable the easier imaging of the vascular diameter. Leakages were sometimes observed due to the presence of small vascular branches, and direct suturing was used to seal these branches.

*Analytical framework*

The analytical framework employed sought to include both the opening angle measurements as well as the pressure-diameter mechanical testing measurements for a more accurate depiction of the vascular wall mechanics.

The schematic diagram of the placental arteries at different configurations is shown in Fig. S1. We defined *State 0* as the stress-free configuration after a radial cut. *R_i_* and *R_o_* were the inner and outer radii at *State 0*. The deformation of the placental arteries from *State 0* to *State 1* was defined as *Deformation 1*. We defined *State 1* as the condition with an intact vessel with no external pressure. Further, we defined *State n* (*n = 2, 3 … n*) as when the vessel was stretched axially back to the *in vivo* length (axial stretch ratio ≈ 1.1), and where a luminal pressure ($P_{n}$) was imposed to expand the vessel lumen. The change from *State 1* to *State n* was defined as *Deformation 2*. $r_{i}^{n}$ and $r_{o}^{n}$ were the inner and outer radii at S*tate n*.


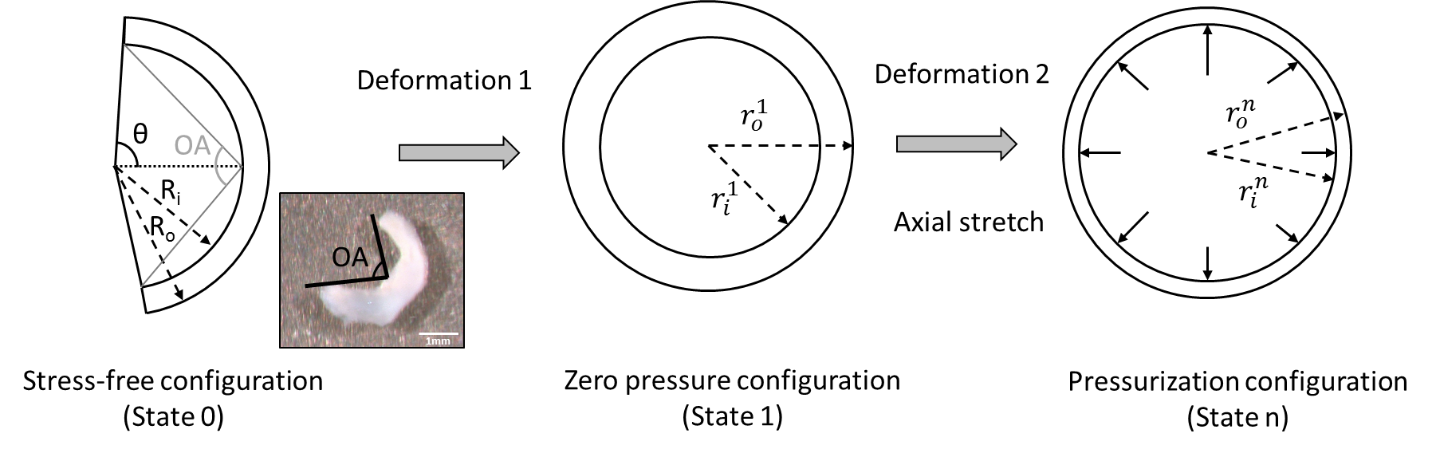


Figure S1 Diagram of the cross-section of a placental artery in different configurations. OA indicates the opening angle, measured between the two lines that connect from the center of the inner sector to the two ends of the inner sector. ϴ indicates the effective angle. R_o_ and R_i_ indicate the outer and inner radii at State 0, as computed from Equation S5. r_o_ and r_i_ indicate the measured outer and inner radii from the experiment at State n. The superscript of r indicates the vessel radius at the n^th^ state.

The mechanical properties of the placental arteries were characterized by a pseudo-strain energy function, *W(****E****)*, as derived by Chuong and Fung ^4^. In this study, we assumed negligible cross stretch (*b_4_* = *b_5_* = *b_6_* =0) and similar stiffness in all three directions (*b_1_* = *b_2_* = *b_3_*).

$\boldsymbol{W}\left( \boldsymbol{E} \right)\boldsymbol{=}\frac{\boldsymbol{c}}{\boldsymbol{2}}\boldsymbol{(}\boldsymbol{e}^{\boldsymbol{Q}\left( \boldsymbol{E} \right)}\boldsymbol{-1)}$ ***Equation S2***

$\boldsymbol{Q(E)=}\boldsymbol{b}_{\boldsymbol{1}}\boldsymbol{E}_{\boldsymbol{\theta}}^{\boldsymbol{2}}\boldsymbol{+}\boldsymbol{b}_{\boldsymbol{1}}\boldsymbol{E}_{\boldsymbol{z}}^{\boldsymbol{2}}\boldsymbol{+}\boldsymbol{b}_{\boldsymbol{1}}\boldsymbol{E}_{\boldsymbol{r}}^{\boldsymbol{2}}$ ***Equation S3***

The material parameters *c* and *b_1_* were the model coefficients describing the material properties. Green strain, *E*, was related to principal stretch ratio, *λ* as stated below, assuming that shear strains were minimal.

$\boldsymbol{E}_{\boldsymbol{i}}\boldsymbol{=}\frac{\boldsymbol{1}}{\boldsymbol{2}}\left( \boldsymbol{\lambda}_{\boldsymbol{i}}^{\boldsymbol{2}}\boldsymbol{-1} \right)\boldsymbol{(i= \theta,r,z)}$ ***Equation S4***

The principal stretch ratios consequent of *Deformation 1* and *Deformation 2* in the three cylindrical axes were computed based on expressions in Table S1.

Table S1. Mathematical expressions for stretch ratios in the three cylindrical axes.

|  | Deformation 1, λ^1^ | Deformation 2, λ^2^ |
| --- | --- | --- |
| Circumferential stretch, *λ_θ_* | $\frac{\pi}{\theta}\frac{\partial r^{1}}{\partial R}$ | $\frac{\partial r^{n}}{\partial r^{1}}$ |
| Axial stretch, *λ_z_* | 1 | 1.11 |
| Radial stretch, *λ_r_* | $\frac{1}{\lambda_{\theta}\lambda_{z}}$ | $\frac{1}{\lambda_{\theta}\lambda_{z}}$ |

The effective angle, *θ*, as defined in Fig. S1, was calculated from the opening angle measurements via trigonometry. Subsequently, *R_i_* and *R_o_* at *State 0* were calculated using Eq. S5, assuming the mid-wall circumference of the vessels were similar at *State 0* and *State 1*.

$\boldsymbol{\theta}\left( \boldsymbol{R}_{\boldsymbol{0}}\boldsymbol{+}\boldsymbol{R}_{\boldsymbol{i}} \right)\boldsymbol{=\pi}\left( \boldsymbol{r}_{\boldsymbol{o}}\boldsymbol{+}\boldsymbol{r}_{\boldsymbol{i}} \right)$ ***Equation S5***

Cauchy stress, **t**, was described by the following equation.

$\boldsymbol{t}= -\boldsymbol{pI}+\boldsymbol{F}\cdot\frac{\boldsymbol{\partial W}(\boldsymbol{E})}{\boldsymbol{\partial E}}\cdot\boldsymbol{F}^{\boldsymbol{T}}$ ***Equation S6***

where *p* is the Langrage multiplier due to incompressibility, ***I*** is the identity matrix and ***F*** is the deformation gradient tensor. At equilibrium, the theoretical pressure, *P* was be computed by using Eq. S7 ^5^.

$\boldsymbol{P}= \int_{\boldsymbol{r}_{\boldsymbol{i}}}^{\boldsymbol{r}_{\boldsymbol{o}}} \boldsymbol{c} ([(\mathbf{1}+\mathbf{2}\boldsymbol{E}_{\boldsymbol{\theta}})(\boldsymbol{b}_{\mathbf{1}}\boldsymbol{E}_{\boldsymbol{\theta}})]-\left[ \left( \mathbf{1}+\mathbf{2}\boldsymbol{E}_{\boldsymbol{r}} \right)\left( \boldsymbol{E}_{\boldsymbol{r}} \right) \right]) \boldsymbol{e}^{\boldsymbol{Q}} \frac{\boldsymbol{dr}}{\boldsymbol{r}}$ ***Equation S7***

where *P* is the luminal pressure, *r_i_* and *r_o_* are the measured outer and inner radii from the experiment and *E* is the Green Strain.

*(B) 1D Modeling of Blood Flow in Umbilical-Placenta Vasculature*

Flow in blood vessels was modeled as an equivalent electrical circuit (Fig. 2a in manuscript), where resistance represented the drag force resisting blood flow and capacitance represented the vascular compliance, which was responsible for the systolic expansion of vessels allowing the vascular system to temporarily store fluid. Flow simulated was idealized to have negligible momentum. Volumetric flow through a segment of vessel was modeled using Eq. S8.

| ${\boldsymbol{Q}_{\boldsymbol{R}}}\boldsymbol{=}\frac{\boldsymbol{\Delta P}}{\boldsymbol{R}}$ | ***Equation S8*** |
| --- | --- |

where *∆P* is the pressure difference between the umbilical artery and vein, and *R*  is the resistance to flow through the vessel. Fluid storage in the compliance (*C*) was modeled using Eq. S9.

| $\boldsymbol{Q}_{\boldsymbol{C}}\boldsymbol{=C}\frac{\boldsymbol{dP}}{\boldsymbol{dt}}$ | ***Equation S9*** |
| --- | --- |

where *Q_C_* is the amount of fluid going into or leaving this compliant vessel, and *dP/dt* is the pressure change with time.

Our model was based on a numerical study of the placental vascular network established with anthropometric measurements comprising of 1 generation of umbilical artery, 10 generations of placental arteries, 1 generation of microvilli, 10 generations of placental veins and 1 generation of umbilical vein ^6^ (Fig. 2a in manuscript). The umbilical artery was modeled with a diameter of 3.2mm ^7^, umbilical coiling index of 0.2coil/cm and a cord length of 0.45m in both normal and IUGR cases ^8^. The umbilical vein diameter was modeled with twice the umbilical artery diameter and a length of 0.45m for both normal and IUGR. Each generation of the placental vessel was prescribed with an average cross-sectional radius, an average segment length, and an approximate density, as tabulated previously ^6^. This allowed the relative resistance and compliance of each generation of vessels to be assigned based on typical vessel size. The resistance of vessels (*R*) was computed using the following equation:

| $\boldsymbol{R=}\frac{\boldsymbol{8}\boldsymbol{\mu l}}{\boldsymbol{\pi}\boldsymbol{r}^{\boldsymbol{4}}}$ | ***Equation S10*** |
| --- | --- |

where *μ* is the dynamic viscosity (0.00336 Pa.s ***^9^***), *l* is the length of vessels and *r* is the radius of vessels.

In both normal and IUGR, the compliance values were simplified to be proportional to the cumulative luminal volume and were adapted from a previously published model ^6^, which obtained data from the literature. For arteries, the compliance varied for a factor of 3.4 through the 11 arterial generations from the largest to the smallest, and the largest vein had a compliance of 8.0 times that of the largest artery, which was consistent with previous study reporting that venous compliance was one order of magnitude higher than arterial compliance ^10^. We had also tested our model with and without consideration of wall thickness factor, and while the magnitude of the Doppler indices changed, the general trends of the results were similar to the results presented in Fig. 6a in the manuscript.

The governing equation for each resistance-compliance pair are as follows (with reference to Fig. 2a in the manuscript):

| $\boldsymbol{P}_{\boldsymbol{i}}\boldsymbol{-}\boldsymbol{P}_{\boldsymbol{i+1}}\boldsymbol{=}\boldsymbol{R}_{\boldsymbol{i}}\boldsymbol{Q}_{\boldsymbol{Ri}}$ | ***Equation S11*** |
| --- | --- |
| $\boldsymbol{Q}_{\boldsymbol{Ci}}\boldsymbol{=}\boldsymbol{C}_{\boldsymbol{i}}\frac{\boldsymbol{d}\boldsymbol{P}_{\boldsymbol{i}}}{\boldsymbol{dt}}$ | ***Equation S12*** |
| $\boldsymbol{Q}_{\boldsymbol{Ri}}\boldsymbol{=}\boldsymbol{Q}_{\boldsymbol{Ri+1}}\boldsymbol{+}\boldsymbol{Q}_{\boldsymbol{Ci+1}}$ | ***Equation S13*** |

where *R_i_* was the resistance of an *i^th^* generation resistor; *C_i_* was the compliance value of an *i^th^* generation capacitor; *Q_Ci_* was the volumetric flow rate going into an *i^th^* generation capacitor; *Q_Ri_* was the volumetric flow rate going through an *i^th^* generation resistor. By assuming every resistor and capacitor of the same generation to be identical, the cumulative conductance, cumulative compliance ($\tilde{C})$, or cumulative volumetric flow rate ($\tilde{Q}_{R}$ or $\tilde{Q}_{C}$) of any particular vascular generation could be calculated by multiplying individual conductance, individual compliance, or individual flow rate with the number of elements in the generation (*N*), as follows. Cumulative resistance ($\tilde{R}$*)* was then computed as the reciprocal of cumulative conductance.

| ${\tilde{\boldsymbol{Q}}}_{\boldsymbol{Ri}}\boldsymbol{=}\boldsymbol{N}_{\boldsymbol{i}}\boldsymbol{Q}_{\boldsymbol{Ri}}$ | ***Equation S14*** |
| --- | --- |
| ${\tilde{\boldsymbol{Q}}}_{\boldsymbol{Ci}}\boldsymbol{=}{\boldsymbol{N}_{\boldsymbol{i}}\boldsymbol{Q}}_{\boldsymbol{Ci}}$ | ***Equation S15*** |
| ${\tilde{\boldsymbol{C}}}_{\boldsymbol{i}}\boldsymbol{=}\boldsymbol{N}_{\boldsymbol{i}}\boldsymbol{C}_{\boldsymbol{i}}$ | ***Equation S16*** |
| $\frac{\boldsymbol{1}}{{\tilde{\boldsymbol{R}}}_{\boldsymbol{i}}}\boldsymbol{=}\boldsymbol{N}_{\boldsymbol{i}}\frac{\boldsymbol{1}}{\boldsymbol{R}_{\boldsymbol{i}}}$ | ***Equation S17*** |

To solve the equations presented, the above equations were written as a differential equation (Eq. S18) and solved with well-established numerical methods.

| $\frac{\boldsymbol{d}}{\boldsymbol{dt}}\left( \begin{matrix} \boldsymbol{P}_{\boldsymbol{0}} \\ \boldsymbol{P}_{\boldsymbol{1}} \\ \begin{matrix} \boldsymbol{\vdots} \\ \boldsymbol{P}_{\boldsymbol{i+1}} \end{matrix} \end{matrix} \right)\boldsymbol{=-}\left( \begin{matrix} \left[ \boldsymbol{P}_{\boldsymbol{0}}\boldsymbol{-}\boldsymbol{P}_{\boldsymbol{1}}\boldsymbol{-}{\tilde{\boldsymbol{R}}}_{\boldsymbol{0}}\boldsymbol{Q}_{\boldsymbol{in}} \right]\boldsymbol{/}{\tilde{\boldsymbol{R}}}_{\boldsymbol{0}}{\tilde{\boldsymbol{C}}}_{\boldsymbol{0}} \\ \begin{matrix} \left[ \boldsymbol{P}_{\boldsymbol{1}}\boldsymbol{-}\boldsymbol{P}_{\boldsymbol{2}}\boldsymbol{-}{\tilde{\boldsymbol{R}}}_{\boldsymbol{1}}{\tilde{\boldsymbol{Q}}}_{\boldsymbol{R}\boldsymbol{0}} \right]\boldsymbol{/}{\tilde{\boldsymbol{R}}}_{\boldsymbol{1}}{\tilde{\boldsymbol{C}}}_{\boldsymbol{1}} \\ \boldsymbol{\vdots} \end{matrix} \\ \left[ \boldsymbol{P}_{\boldsymbol{i+1}}\boldsymbol{-}\boldsymbol{P}_{\boldsymbol{i}\boldsymbol{+}\boldsymbol{2}}\boldsymbol{-}{\tilde{\boldsymbol{R}}}_{\boldsymbol{i+1}}{\tilde{\boldsymbol{Q}}}_{\boldsymbol{Ri}} \right]\boldsymbol{/}{\tilde{\boldsymbol{R}}}_{\boldsymbol{i+1}}{\tilde{\boldsymbol{C}}}_{\boldsymbol{i+1}} \end{matrix} \right)$ | ***Equation S18*** |
| --- | --- |

where *P_0_* was the pressure at the inlet of the umbilical arteries which was set at 50/25 mmHg (systolic/diastolic) for the normal case ^11^ and 80/25 mmHg in the IUGR case ^12^. *P_i+2_* was the pressure in the umbilical vein and was prescribed as a time-independent constant at 5.3 mmHg ^13^. *Q_in_* was the cumulative flow rate at the inlet of the two umbilical arteries. The subscript zero in Eq. S18 referred to umbilical artery. Eq. S18 was solved using a built-in Ordinary Differential Equation (ODE) solver in MATLAB® (Mathworks Inc., Natick, USA) and flow rates in the placental circulation were computed with Eq. S19.

| $\left( \begin{matrix} \boldsymbol{Q}_{\boldsymbol{R}\boldsymbol{1}} \\ \begin{matrix} \boldsymbol{Q}_{\boldsymbol{R}\boldsymbol{2}} \\ \boldsymbol{\vdots} \\ \boldsymbol{Q}_{\boldsymbol{R}\boldsymbol{i}} \end{matrix} \end{matrix} \right)\boldsymbol{=}\left( \begin{matrix} \boldsymbol{(}\boldsymbol{P}_{\boldsymbol{1}}\boldsymbol{-}\boldsymbol{P}_{\boldsymbol{2}}\boldsymbol{)/}\boldsymbol{R}_{\boldsymbol{1}} \\ \boldsymbol{(}\boldsymbol{P}_{\boldsymbol{2}}\boldsymbol{-}\boldsymbol{P}_{\boldsymbol{3}}\boldsymbol{)/}\boldsymbol{R}_{\boldsymbol{2}} \\ \begin{matrix} \boldsymbol{\vdots} \\ \boldsymbol{(}\boldsymbol{P}_{\boldsymbol{i}}\boldsymbol{-}\boldsymbol{P}_{\boldsymbol{i}\boldsymbol{+1}}\boldsymbol{)/}\boldsymbol{R}_{\boldsymbol{i}} \end{matrix} \end{matrix} \right)$ | ***Equation S19*** |
| --- | --- |

**RESULTS**

Table S2 Patients’ characteristics. Data are presented as mean ± standard deviation. GA – Gestational age. **^*^** P < 0.05 vs normal group. **^†^** P < 0.05 vs IUGR group. ^a^ Anthropometric Analysis. ^b^ Pressure-Diameter Mechanical Testing. Student two-tailed t-test and ANOVA test were carried out.

|  | Normal | IUGR | Severe IUGR |
| --- | --- | --- | --- |
| Delivery GA (Weeks) | ^a^ 39.1±1.3  ^b^ 39.3±1.6 | ^a^ 38.2±0.4  ^b^ 37.7±0.9 | ^b^ 35.8±2.9 **^*^** |
| Birthweight (kg) | ^a^ 3.203±0.380  ^b^ 3.272±0.500 | ^a^ 2.396±0.207 **^*^**  ^b^ 2.428±0.276 **^*^** | ^b^ 1.746±0.467 **^*^** |
| RI | ^a^ 0.61±0.06  ^b^ 0.55±0.07 | ^a^ 0.57±0.07  ^b^ 0.58±0.05 | ^b^ 0.72±0.02 **^*†^** |
| RI (centile) | ^a^ 57.5±24.7  ^b^ 40.3±24.1 | ^a^ 46.0±25.6  ^b^ 54.0±20.7 | ^b^ 94.3±3.5 **^*†^** |
| PI | ^a^ 0.92±0.16  ^b^ 0.80±0.12 | ^a^ 0.84±0.18  ^b^ 0.87±0.10 | ^b^ 1.27±0.07 **^*†^** |
| PI (centile) | ^a^ 60.1±26.1  ^b^ 40.2±20.4 | ^a^ 45.6±30.6  ^b^ 59.3±21.8 | ^b^ 98.3±1.3**^*†^** |

**REFERENCES**

1 Junaid, T. O., Bradley, R. S., Lewis, R. M., Aplin, J. D. & Johnstone, E. D. Whole organ vascular casting and microCT examination of the human placental vascular tree reveals novel alterations associated with pregnancy disease. *Scientific Reports* **7**, 4144 (2017).

2 Gordon, Z. *et al.* Anthropometry of fetal vasculature in the chorionic plate. *Journal of Anatomy* **211**, 698-706, doi:10.1111/j.1469-7580.2007.00819.x (2007).

3 Stemper, B. D. *et al.* Mechanics of fresh, refrigerated, and frozen arterial tissue. *The Journal of surgical research* **139**, 236-242, doi:10.1016/j.jss.2006.09.001 (2007).

4 Chuong, C. J. & Fung, Y. C. On residual stresses in arteries. *Journal of biomechanical engineering* **108**, 189-192 (1986).

5 Wang, R. & Gleason, R. L., Jr. A mechanical analysis of conduit arteries accounting for longitudinal residual strains. *Ann Biomed Eng* **38**, 1377-1387, doi:10.1007/s10439-010-9916-6 (2010).

6 Clark, A. R., Lin, M., Tawhai, M., Saghian, R. & James, J. L. Multiscale modelling of the feto-placental vasculature. *Interface focus* **5**, 20140078, doi:10.1098/rsfs.2014.0078 (2015).

7 Saw, S. N., Dawn, C., Biswas, A., Mattar, C. N. Z. & Yap, C. H. Characterization of the in vivo wall shear stress environment of human fetus umbilical arteries and veins. *Biomechanics and modeling in mechanobiology* **16**, 197-211 (2017).

8 Georgiadis, L. *et al.* Umbilical cord length in singleton gestations: A Finnish population-based retrospective register study. *Placenta* **35**, 275-280, doi:<https://doi.org/10.1016/j.placenta.2014.02.001> (2014).

9 Riopel, L., Fouron, J. C. & Bard, H. Blood viscosity during the neonatal period: the role of plasma and red blood cell type. *J Pediatr* **100**, 449-453 (1982).

10 Gelman, S. Venous function and central venous pressure: a physiologic story. *ANESTHESIOLOGY-PHILADELPHIA THEN HAGERSTOWN-* **108**, 735 (2008).

11 Struijk, P. C. *et al.* Blood pressure estimation in the human fetal descending aorta. *Ultrasound in obstetrics & gynecology : the official journal of the International Society of Ultrasound in Obstetrics and Gynecology* **32**, 673-681, doi:10.1002/uog.6137 (2008).

12 Miyashita, S. *et al.* Measurement of internal diameter changes and pulse wave velocity in fetal descending aorta using the ultrasonic phased-tracking method in normal and growth-restricted fetuses. *Ultrasound Med Biol* **41**, 1311-1319, doi:10.1016/j.ultrasmedbio.2014.12.008 (2015).

13 Weiner, C. P. *et al.* Normal values for human umbilical venous and amniotic fluid pressures and their alteration by fetal disease. *American Journal of Obstetrics & Gynecology* **161**, 714-717, doi:10.1016/0002-9378(89)90387-6 (1989).
